# A sustainable approach to biobased porous organic frameworks and their composites

**DOI:** 10.1039/d5sc08149a

**Published:** 2025-12-17

**Authors:** Michelle Åhlén, Samson Afewerki, Chao Xu

**Affiliations:** a Division of Nanotechnology and Functional Materials, Department of Materials Science and Engineering, Ångström Laboratory, Uppsala University SE-751 21 Uppsala Sweden chao.xu@angstrom.uu.se; b Department of Chemistry, College of Science, United Arab Emirates University Al Ain Box 15551 United Arab Emirates

## Abstract

Porous organic frameworks (POFs) represent a diverse group of porous materials that have gained significant recognition in the last couple of decades. Their synthetic diversity and modular structure enable the construction of extended network structures with tailored pore architectures and chemical functionalities. Composed of functional organic monomers, the molecular building blocks of POFs are predominantly derived from fossil fuel-based feedstock, which poses a significant challenge to the long-term sustainability of these materials. Recent advances in the development of biobased POFs and their composites from renewable and natural precursors offer a promising route to carbon-neutral and cost-efficient synthesis of these framework materials. This perspective highlights emerging synthetic strategies for constructing biobased POFs and their composites from renewable organic monomers derived from biomass, such as lignin and cellulose. We outline opportunities and key challenges in the field, and propose a pathway for realizing a closed-loop system for fabricating the next generation of sustainable functional POF materials.

## Abstract 2

The use of biobased monomers and biopolymers for the fabrication of porous organic frameworks and their composites provides a sustainable approach to developing functional porous materials and their practical applications.

## Graphical abstract

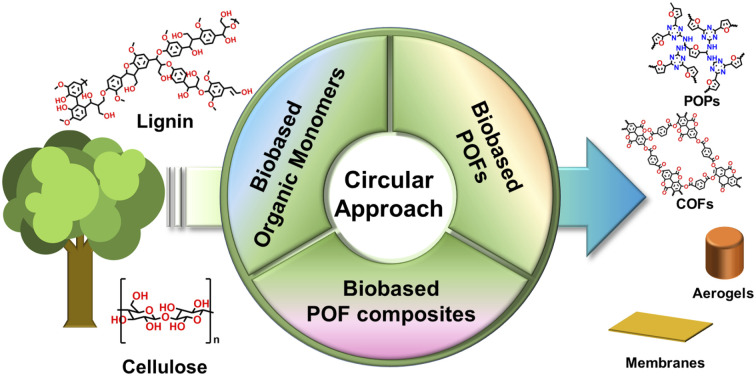

## Introduction

Porous organic frameworks (POFs), including hyper crosslinked polymers (HCPs), porous organic polymers (POPs), and covalent organic frameworks (COFs), are emerging families of functional porous materials constructed by linking purely organic building blocks through strong covalent bonds to form extended porous networks. HCPs and POPs are typically amorphous with limited long-range order, while COFs are crystalline, featuring well-defined, ordered framework structures with uniform porous channels. Both materials generally exhibit high surface areas (up to several thousand square meters per gram), along with tunable pore sizes and excellent physicochemical stability. In addition, they offer great synthetic versatility, as a wide variety of organic linkers can be used in different polymerization reactions. Furthermore, the rich organic functionality on their surfaces or within the pores allows for easy post-synthetic modification and functionalization.^1–4^ These features make POFs highly attractive for diverse applications, including gas storage and separation,^5,6^ catalysis,^7–10^ energy storage,^11,12^ and environmental remediation.^13,14^

Most reported POFs are synthesized from synthetic monomers containing aromatic or heterocyclic units, which are primarily derived from fossil fuel-based feedstocks. The preparation of these monomers typically requires multiple synthetic steps and harsh reaction conditions, often resulting in high manufacturing costs and a significant environmental footprint. In particular, monomers bearing more than two reactive functional groups, which are essential for constructing two- (2D) or three-dimensional (3D) extended frameworks with permanent porosity, are often very expensive due to their synthetic complexity, with costs reaching several thousand dollars per gram. Consequently, the large-scale production of POFs using such monomers remains both challenging and impractical.^15–17^ Furthermore, most POFs are obtained as insoluble and infusible powders, which cannot be processed into freestanding forms such as membranes, granules, or monoliths using conventional solution or melt-processing techniques employed for traditional polymers.^18^ Although organic binders can be introduced to shape POF powders, this approach adds inactive mass and volume, and may also block the intrinsic porous channels, thereby reducing accessible surface area and porosity.^19^ These limitations in green, scalable synthesis and processability have significantly hindered the practical deployment of POFs.

Biomass is a sustainable and renewable resource with great potential for value-added applications. The annual global production of biomass is estimated to be 170 billion metric tons per year.^20^ Proper utilization of biomass is strategically important from both economic and environmental perspectives, offering a feasible solution to the ongoing energy crisis. Depending on its source, biomass contains abundant aromatic and heterocyclic units that can be valorized into high-value chemicals and functional materials. For instance, lignin, a major component of lignocellulosic biomass, is rich in aromatic structures. Valuable aromatic compounds such as vanillin, syringaldehyde, and guaiacol can be extracted from lignin through controlled depolymerization and catalytic conversion processes.^21^ In the sugar industry, hemicellulose-derived pentoses (*e.g.*, xylose) can be converted into furfural compounds *via* acid-catalyzed dehydration, producing key intermediates such as furfural and 5-hydroxymethylfurfural (HMF).^22^ These bio-derived aromatic and heterocyclic compounds can serve as potential monomers or building blocks for the synthesis of POFs. In particular, HMF derived from hemicellulose or lignin can, after minor synthetic manipulations, generate dialdehyde and tri-aldehyde compounds, which are suitable for biobased POF preparation.^23,24^ In addition, cellulose, the most abundant biopolymer on Earth, has traditionally been used in the paper and packaging industries. Beyond these conventional applications, cellulose holds great promise in advanced materials processing. It can act as a template, scaffold, or substrate to support and shape a wide variety of functional nanomaterials, such as conducting polymers,^25^ inorganic nanoparticles,^26^ and metal–organic frameworks (MOFs),^27–29^ enabling the fabrication of flexible films, aerogel, and hydrogels for energy, environmental, and biomedical applications. Similarly, cellulose could potentially facilitate the processing of POFs into freestanding and functional forms for practical use. Therefore, the use of biomass for both the synthesis and engineering of POFs presents a promising strategy to address current challenges in the scalable production, functional shaping, and application development of POFs.

In this perspective, we summarize recent progress in the development of biobased POFs, with a focus on their synthesis from renewable monomers and the use of biopolymers for structural engineering. We also highlight the applications of these POFs and their biopolymer composites. Finally, we present our views on future directions for advancing the synthesis and engineering of biobased POFs, their practical implementation across various applications, and the associated challenges and opportunities.

## The chemistry and synthesis of POFs

Interconnecting small organic building blocks into linear one-dimensional (1D) or branched structures *via* addition or condensation reactions has long been used to synthesize conventional polymers at an industrial scale, yielding mechanically robust yet poorly ordered and dense structures.^30–32^ In contrast, the network structure of POFs arises from the chemical interlinking of multifunctional and geometrically constrained monomers or polymeric chains, which provides a topological basis for the formation of 2D and 3D networks.^33^ In particular, transforming rigid polymers into extended and highly interconnected architectures by chemical crosslinking has served as a facile pathway for the construction of HCPs since the 1970s, leading to their establishment as the first commercialized POF.^34^ The development of HCPs has since progressed to include network structures formed through both Friedel–Crafts alkylation of polymers and the self-condensation of multifunctional monomers.^35^ In particular, the linking of monomers with various organic functionalities has expanded beyond HCPs, resulting in diverse POFs with a rich linkage chemistry, including olefin,^16,36–38^ alkyl,^39–41^ hydrazone,^42–45^ β-ketoenamine,^46–49^ and imide linkages.^50–53^ However, imine-linked frameworks arguably constitute the largest and most common class of POF.^54,55^ Derived from Schiff base condensation reactions between aldehydes and amines, the formation of kinetic (amorphous) or thermodynamic (crystalline) products is significantly influenced by both the position and reactivity of the functional groups on the monomers, as well as on the reaction media and conditions.^56,57^ Strict synthetic control has therefore typically been required, especially for COFs whose ordered structures depend on carefully tuned imine-exchange rates.^58,59^ For instance, one of the earliest imine-linked POFs was reported in 2009 by Uribe-Romo *et al.*^60^ from the acetic acid-catalyzed reaction between terephthalaldehyde (TPA) and tetra-(4-anilyl)methane (TAM) in dioxane at 120 °C. The chemical interlinking of the linear TPA and tetrahedral TAM monomers resulted in the formation of an ordered 5-fold interpenetrated 3D structure, which exhibited a surface area exceeding 1300 m^2^ g^−1^. A year later, Pandey *et al.*^61^ presented a series of POP materials derived from 1,3,5-triformylbenzene (TFB) and different diamines with surface areas reaching up to 1500 m^2^ g^−1^, representing a significant milestone in the development of highly porous amorphous imine-linked POFs. The seminal work by these authors marked the beginning of research into imine-linked POFs, and the field has since rapidly developed to include a wide library of POF structures and contributed to advances in related areas such as single-crystal growth,^62–65^ material processing,^66–69^ and green synthetic approaches.^17,46,70,71^

The sustainable synthesis of POFs, in particular, has gained significant attention in recent years, driven by the drawbacks of traditional synthetic procedures that rely on organic solvents and harsh reaction conditions. Green solvents, such as water, acetic acid, γ-butyrolactone, and supercritical CO_2_,^17,69,70,72,73^ have successfully been utilized to tune the polymerization reaction of various organic monomers, resulting in the construction of POFs with comparable or superior properties to their solvothermal counterparts.^70,74^ Furthermore, solvent-free mechanochemical approaches have also shown that COFs can be constructed in the absence of liquid media.^46,74^ These advancements have set a new precedent for the environmentally friendly synthesis of POFs.

### POFs synthesized from biobased monomers

**Fig. 1:**
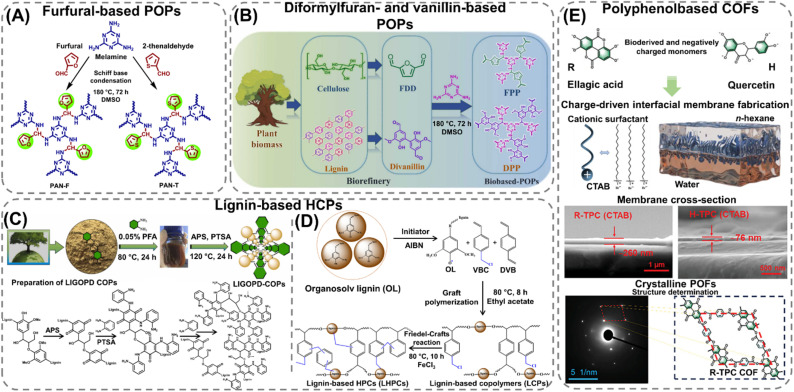
Synthesis scheme of various biobased POFs: Schiff base POPs (A) polyaminal-furfural (PAN-F) and polyaminal-2-thenaldehyde (PAN-T), constructed from the Schiff base condensation of biomass-derived furfural and melamine at solvothermal conditions. Reproduced from ref. 76 with permission from the Royal Society of Chemistry, (B) FPP and DPP POPs synthesized from melamine, dihydrofuran (FDD or DFF), and divanillin, respectively, at solvothermal conditions. Reproduced with permission from ref. 77. Copyright 2022, Elsevier. (C) Lignin-based HCPs (LIGOPD-COPs) prepared from a one-pot reaction between natural lignin and 1,2-diaminobenzene (OPD) in the presence of paraformaldehyde (PFA), ammonium persulfate (APS), and *p*-toluenesulfonamide (PTSA) at hydrothermal conditions. Reproduced with permission from ref. 85. Copyright 2023, Elsevier. (D) Synthesis of lignin-based HCPs *via* Friedel–Crafts alkylation of lignin-based copolymers (LCPs). The LCPs were prepared by graft polymerisation of organosolv lignin (OL) with 4-vinylbenzyl chloride (VBC) and divinylbenzene (DVB) in the presence of isobutyronitrile (AIBN). Reproduced with permission from ref. 86. Copyright 2021, American Chemical Society. (E) Ester-linked COFs synthesized from terephthaloyl chloride (TPC) with ellagic acid (R) or quercetin (H), and the corresponding COF membranes fabricated *via* charge-driven interfacial synthesis in the presence of cetyl trimethyl ammonium bromide (CTAB). SEM images showing the film thickness of the COF membranes and the selected-area electron diffraction (SAED) pattern revealing the highly crystalline nature and crystal structure of R-TPC COF. Reproduced with permission from ref. 91. Copyright 2024, Wiley-VCH.

Naturally occurring polyphenols represent a group of molecular compounds found in a wide range of plant-based sources, including fruits, nuts, seeds, and wood. The aromatic backbone of these molecules, richly decorated with hydroxyl and ester groups, exhibits many of the structural features required of monomers for the fabrication of POFs with high porosities (Table 1). For instance, mono- and dialdehyde compounds such as furfural, diformylfuran (DFF), and vanillin (Fig. 1A and B) can be obtained from the depolymerization and conversion of biomass, and have been used for the construction of amorphous POP materials.^75–78^ The reaction between the aldehyde compounds and diamines (*e.g.*, *m*-phenylenediamine and benzidine)^75^ or triamines (*e.g.*, benzene-1,3,5-triamine and melamine)^76–78^ has been found to result in the formation of porous imine-linked HCPs and POPs with surface areas ranging from 19 to 1095 m^2^ g^−1^. The geometry of the diamine compounds was found to have an observable effect on both the porosity and morphology of the POPs—bent monomers such as *m*-phenylenediamine and 2,6-pyridinediamine enabled the formation of branched network structures, which prevented the dense packing of polymer chains and contributed to the growth of spherical nanoparticles.^17^ In contrast, linear diamine monomers yielded tightly packed and less porous network structures, resulting in the formation of dendrite-shaped particle aggregates or sheets.^75^ In a similar manner, Xiong *et al.*^79^ utilized a carboxylate analogue of DFF, 2,5-furandicarboxylic acid (FDCA), together with melamine to fabricate an amide-linked POP in a mixture of chloroform, ethyl chloroformate, dimethyl sulfoxide (DMSO), and triethylamine at 145 °C. Compared to the DFF-derived POP, which had a low surface area of 19 m^2^ g^−1^ and a pore size of 19 nm,^78^ the porosity of the FDCA-based POF was significantly higher (208 m^2^ g^−1^ and 6.4 nm), due to the increased flexibility of the amide linkages. The nitrogen-decorated framework could furthermore be used to selectively capture mercury ions from water, showing the capabilities of the POP to act as a sustainable and affordable sorbent for heavy metal removal. Additionally, Liu *et al.*^77^ and Asadi *et al.*^80^ presented a two-step process for the preparation of vanillin-based POFs, which exhibited high porosities ranging from 378 to 514 m^2^ g^−1^ and pore sizes of 3.0 to 3.5 nm, that showed promising performances as functional sorbents for organic pollutants^77^ and as biocompatible drug delivery vehicles.^80^

Moreover, the depolymerization of technical lignin can produce carboxylic and aldehydic compounds as well as a wide range of small phenolic molecules, such as *p*-methoxyphenol and syringaldehyde, which can be used for fabricating POFs.^81–84^ For instance, by chemically crosslinking catechol (Ccol), 2,3-naphthalenediol (Ntdiol), or 4-methyl-catechol (Mcol) with formaldehyde dimethyl acetal (FDA) *via* Friedel–Crafts alkylation, Zhao *et al.*^83^ successfully prepared extended catechol-based HCPs. The materials exhibited highly crosslinked structures and surface areas ranging from 34 to 665 m^2^ g^−1^. Furthermore, lignin itself can also be utilized as a phenol-rich building block for the construction of POFs.^85–89^ In particular, Li *et al.*^85^ highlighted a series of unique HCPs obtained from a one-pot reaction between natural lignin and 1,2-diaminobenzene (OPD) in water at 80 °C (Fig. 1C). The reaction was carried out at basic conditions and in the presence of paraformaldehyde (PFA), ammonium persulfate (APS), and *p*-toluenesulfonamide (PTSA), which were used to oxidize the hydroxyl and methoxy groups on the lignin molecules and drive the crosslinking reaction between amine and quinone, as well as quinone carbonyl groups. Although the HCPs exhibited relatively low surface areas (<35 m^2^ g^−1^), they were found to be capable of adsorbing and removing phytochromes from vegetables. This effect could be attributed to the strong hydrophobic interactions between the aromatic structure of the HCPs and the targeted chemicals, as well as the relatively large pore size (8.6 nm) of the materials that likely facilitated the adsorption of these bulky molecules. Furthermore, Liu *et al.*^86^ employed a free radical copolymerization strategy to enhance the porosity of organosolv lignin-based POFs by synthesizing a 4-vinylbenzyl chloride (VBC)- and divinylbenzene (DVB)-crosslinked lignin-based copolymer from which HCPs were produced *via* Friedel–Crafts alkylation (Fig. 1D). The HCPs possessed hierarchical network structures and high porosities, with surface areas ranging from 1076 to 1500 m^2^ g^−1^ and pore volumes exceeding 1 cm^3^ g^−1^. The high porosity, along with the presence of carbonyl- and hydroxy-groups on the pore surfaces, could facilitate the capture of iodine vapor.

Although lignocellulosic biomass can be found in abundance and represents a significant by-product of the pulping industry, other polyphenolic compounds can also be extracted from fruits and vegetables.^90–93^ In particular, ellagic acid is naturally released in ripened fruits such as pomegranates and strawberries.^94^ The molecule has a rigid and symmetrical structure that makes it a highly suitable building block for framework materials.^91,95–97^ For instance, Du *et al.*^91^ reported the fabrication of two ester-linked COF membranes, obtained from the surfactant-mediated interfacial synthesis of terephthaloyl chloride (TPC) and the bio-derived phenolic monomers ellagic acid or quercetin in *n*-hexane/water (Fig. 1E). The authors achieved a rapid membrane formation due to electrostatic interaction between the cationic cetyl trimethyl ammonium bromide (CTBA) surfactants, partitioned at the liquid interface, and the negatively charged biomolecules. The resulting COF films, which formed in a matter of seconds, exhibited a 75–250 nm film thickness and possessed excellent nanofiltration properties thanks to their well-defined crystal structure. Similarly, Thakkar *et al.*^92^ utilized ellagic acid and 1,4-phenylene diisocyanate (PDI) or methylene diphenyl diisocyanate (MDI) to construct amide-linked COFs in acetonitrile *via* sonication at 35 °C. The frameworks possessed moderate crystallinity and low surface areas and pore volumes ranging from 14 to 35 m^2^ g^−1^ and 0.08 to 0.20 cm^3^ g^−1^, respectively. This effect likely originated from the increased structural flexibility of the amine monomers, as well as the bulky structure of ellagic acid, which may have contributed to the formation of dense frameworks. Beyond ellagic acid, the construction of porous HCPs from diverse biobased polyphenolic compounds has been exemplified by Björnerbäck *et al.*,^93^ who carried out the crosslinking of various biobased molecules, such as quercetin and tannic acid, *via* Friedel–Crafts alkylation in sulfolane at reflux conditions. The formed HCPs exhibited high porosities, with surface areas ranging from 600 to 1300 m^2^ g^−1^ and pore volumes up to 1 cm^3^ g^−1^, reflecting their interconnected network structures and heterogeneous pore surfaces arising from variations in the phenolic content within the materials.

### Biopolymers and their role in structuring functional materials

Nature offers a vast repertoire of diverse biopolymers, produced by both plants and living organisms, that serve key roles in essential processes such as structural support and energy storage. The unique physicochemical and mechanical properties of these macromolecules are derived from their chemical structure. For instance, the complex and crosslinked structure of lignin is richly decorated with carbonyl, methoxy, and hydroxyl groups that can serve as reactive sites for chemical modifications and grafting. Similarly, linear polysaccharide compounds, such as chitosan, cellulose, and alginate, contain amino, carboxylate, and hydroxyl groups that can be used for the same purpose.^98–100^ Beyond their surface chemistry, the extended polymeric chains endow the macromolecules with exceptional strength and elastic properties, imparting them with notable durability. These abilities have enabled many biopolymers to act as functional binders, allowing for the processing of otherwise difficult-to-handle materials, as well as imbuing their composite structures with improved mechanical strength and flexibility.^101^ For instance, hierarchically porous aerogels of bacterial cellulose (BC) and two MOFs were reported by Ma *et al.*^102^*via* a rapid interfacial growth of the framework materials on the polymer chains. The metal ions were anchored onto the hydroxyl groups of the cellulose, which acted as a scaffold from which the framework structures could grow, yielding a 43 to 55 wt% MOF loading. The resulting BC@MOF composites inherited both the porosity of the MOF materials and the mechanical properties of the cellulose, yet exhibited superior adsorption properties compared to their pristine counterparts due to the hierarchical pore structure of the aerogels. Similarly, cellulose and other lignocellulosic biomass have been used as substrates to process a wide range of functional materials from zeolites^103^ and MXenes (Ti_3_C_2_T_*x*_)^104^ to graphene^105^ and carbon nanotubes.^106,107^ The viscoelastic and rheological properties of many biopolymeric gels also offer significant advantages when shaping composite structures, particularly when using processing techniques that require controlled material flow and placement, such as extrusion-based additive manufacturing techniques. For instance, 3D-printed scaffolds were fabricated by Sultan *et al.*^108^ from hybrid inks of sodium alginate and MOF-decorated carboxylate CNF. The scaffolds were shaped into 2 × 2 × 2 cm cubes featuring 1 mm pores spaced approximately 1.5 mm apart. The deprotonated carboxylic chain on the alginate polymer enabled the ionic crosslinking of the printed structures by CaCl_2_, resulting in stable scaffolds with appreciable durability.

### Biopolymer-based POF composites

**Fig. 2:**
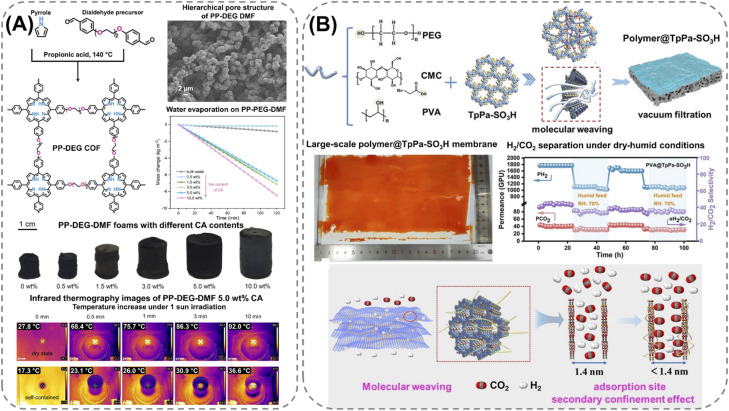
Fabrication processes and properties of cellulose-derived biopolymer composites: (A) 3D composite foams of cellulose acetate (CA) and a porphyrin-based polyCOF (PP-DEG) possessing a crosslinked hierarchically porous structure and efficient photothermal conversion properties, as shown by the high water evaporation rate of the material under 1 sun irradiation, which is driven by the increase in surface temperature, as shown by infrared thermography images of the PP-DEG-DMF CA foam. Reproduced with permission from ref. 109. Copyright 2024, CC BY. (B) Molecularly woven polymeric composite membrane, consisting of polyethylene glycol (PEG), polyvinyl alcohol (PVA), or carboxymethyl cellulose (CMC), and a sulfonate COF (TpPa-SO_3_H) possessing a constricted pore size and a sulfonated- and hydroxyl-decorated pore surface capable of separating H_2_ from CO_2_. Reproduced with permission from ref. 110. Copyright 2024, Wiley-VCH.

**Fig. 3:**
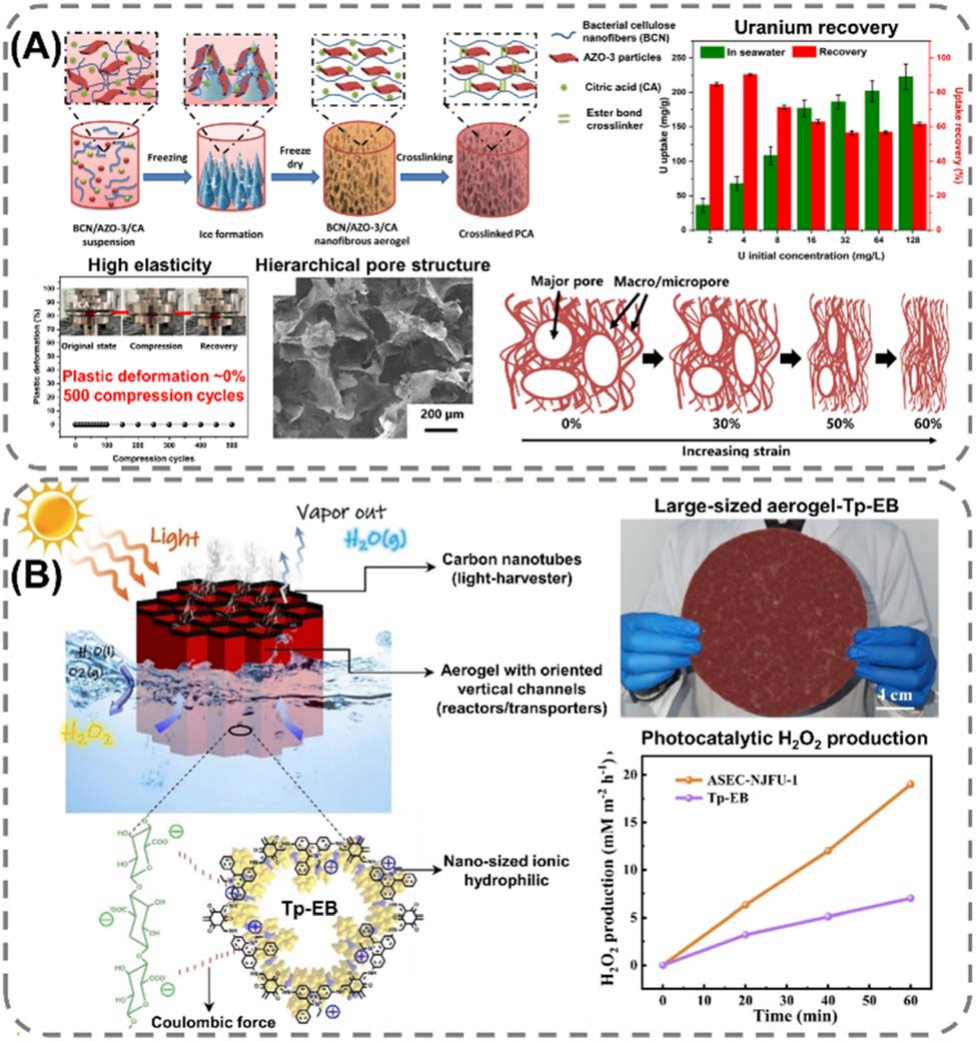
Synthesis process and application of cellulose composite aerogels: (A) highly elastic ester-crosslinked aerogel composed of bacterial cellulose nanofibers (CNF) and 2D COF (AZO-3), possessing a hierarchical porosity, exceptional elasticity, and promising properties for extracting and recovering uranium from seawater. Reproduced with permission from ref. 111. Copyright 2024, Elsevier. (B) Composite aerogel (ASEC-NJFU-1), consisting of nanocellulose, carbon nanotubes, and an ionic COF (Tp-EB), capable of acting as an artificial solar energy converter (ASEC), generating both fresh water and producing hydrogen peroxide by photocatalysis. Reproduced with permission from ref. 112. Copyright 2025, Wiley VCH.

**Fig. 4:**
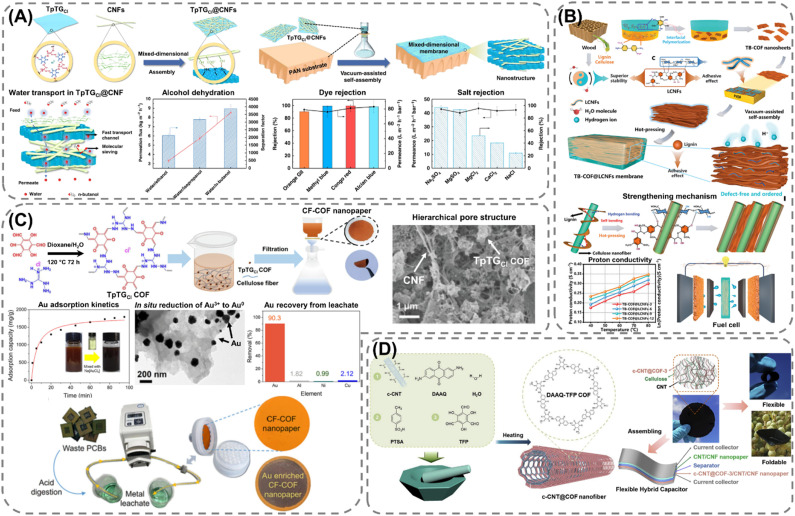
Synthetic methods for fabricating cellulose–COF composite membranes: (A) PAN-supported membrane of cellulose nanofibers (CNF) and an ionic COF (TpTG_Cl_) fabricated *via* vacuum-assisted self-assembly, exhibiting molecular sieving properties and high rejection rates of alcohols, dyes, and salts. Reproduced with permission from ref. 113. Copyright 2019, CC BY. (B) Lignocellulosic nanofibrils (LCNF)-based membrane containing a sulfonated COF (TB-COF) constructed *via* vacuum-assisted self-assembly and hot-pressing, exhibiting excellent mechanical strength induced from the hot-pressing treatment, which encourages the formation of hydrogen-bonding between the lignin nanoparticles and COF. The composite membrane possesses promising proton-conducting properties and suitable mechanical strength for integration into next-generation energy storage devices. Reproduced with permission from ref. 114. Copyright 2023, Wiley-VCH. (C) CNF-based nanopaper formed *via* vacuum-assisted self-assembly and utilized for the recovery of gold from electronic waste acidic leachate. Reproduced with permission from ref. 115. Copyright 2023, CC BY. (D) Composite membrane composed of CNF and a redox-active COF (DAAQ-TFP COF) grown on carboxylate multi-walled carbon nanotubes (c-CNTs), fabricated *via* vacuum self-assembly and exhibiting a flexible structure and good electrochemical performance, enabling its integration into a flexible hybrid capacitor for energy storage. Reproduced with permissions from ref. 116. Copyright 2021, CC BY.

Extending the utilization of biopolymers to the processing of POFs presents a promising strategy toward the fabrication of fully organic and sustainable composite materials. Shaping these materials into, *e.g.*, membranes, aerogels, monoliths, and hydrogels constitutes a crucial step for their integration into industrial applications and for their practical use. The functional groups present on the polymers enable the attachment of POF particles to the polymeric chains, as well as direct interfacial growth of the framework materials on the chains. For instance, cellulose derivatives such as cellulose acetate (CA) and carboxymethyl cellulose (CMC) can serve as functional binders to facilitate the formation of molecularly-woven composite structures. In particular, Hu *et al.*^109^ and Tian *et al.*^110^ presented the fabrication of 3D COF foams (Fig. 2A) and freestanding COF membranes (Fig. 2B) *via* self-assembly of the framework materials with CA and CMC, respectively. In particular, the presence of CA in the porphyrin-based COF foams^109^ not only increased the hydrophilicity of the composite structures but also significantly improved their photothermal conversion properties, facilitating high water evaporation rates under 1 sun irradiation (Fig. 2A). For the CMC-based sulfonated COF membranes,^110^ the inclusion of CMC and other hydrophilic polymers, such as polyvinyl alcohol (PVA) and polyethylene glycol (PEG), promoted the formation of large-scale membranes (Fig. 2B). The polymers also facilitated the development of molecularly woven composite structures, which was driven by hydrogen bonding, van der Waals, and electrostatic interactions between the functional groups on the polymer chains and the interior pore surface of the COF. Constriction of the pore size from the intrusion of these polymers, as well as secondary confinement effects induced by specific guest–host interactions, further improved the H_2_/CO_2_ separation properties of the membranes, making them highly promising for the separation of small gas molecules. Furthermore, composite aerogel structures of COFs and nanocellulose were fabricated by Fan *et al.*^111^ and Xu *et al.*^112^ by anchoring particles of the materials to the cellulose chains *via* hydrogen bonding and coulombic forces, producing composite structures with excellent elastic properties and mechanical strength, respectively (Fig. 3A and B). The aerogels achieved ultra-low densities ranging from 4.5 to 27.5 mg cm^−3^ and possessed micrometer-sized pores, which allowed for efficient transfer of water molecules or metal ions within the structures and enabled the composites to be utilized for the extraction and photocatalytic reduction of UO_2_^2+^ to UO_2_,^111^ and the conversion of H_2_O to H_2_O_2_.^112^ Similar synthetic strategies were used by Yang *et al.*,^113^ Zhu *et al.*,^114^ Xu *et al.*,^115^ and Kong *et al.*^116^ to fabricate composite membranes (Fig. 4A–D). By subjecting suspensions of COF-anchored cellulose or lignocellulosic nanofibrils (LCNF) to filtration instead of freeze-drying, the authors achieved free-standing and flexible membranes rather than aerogels. For instance, Yang *et al.*^113^ utilized an ionic COF, TpTG_Cl_ (where Tp = 1,3,5-triformylphloroglucinol and TG_Cl_ = triaminoguanidinium chloride), to attach the framework material onto TEMPO-oxidized CNF *via* electrostatic interactions between the positively charged guanidine groups on the COF and the negatively charged carboxylate groups on the CNF (Fig. 4A). The hierarchically fibrous structure of the membranes could subsequently be utilized for membrane separation, where the active COF structure and the lamellar structure of the composite enabled a high rejection rate of dyes, salts, and alcohols.^113^ Moreover, CNF-composite COF films have also been shown to possess suitable chemical functionalities and pore structures for a variety of advanced applications, such as proton conduction^114^ and noble metal recovery from aqueous media.^115^ In particular, Zhu *et al.*^114^ fabricated composite membranes of exceptional durability by linking COF sheets to LCNFs *via* vacuum-assisted self-assembly and hot-pressing (Fig. 4B). The hot-pressing treatment was found to play a pivotal role in increasing the mechanical strength of the composite by promoting the dispersibility of lignin nanoparticles in the membranes and facilitating the formation of strong hydrogen bonds with the COF nanosheets and the lignin particles themselves. The increased lamellar interaction between neighboring COF nanosheets furthermore led to the formation of a well-ordered pore structure capable of supporting fast proton transport, rendering the composite material highly interesting for fuel cell applications. Additionally, Xu *et al.*^115^ and Kong *et al.*^116^ constructed COF nanopapers *via* vacuum-assisted self-assembly of COFs and CNFs (Fig. 4C and D). The membranes displayed a hierarchical pore structure derived from the aggregation of cellulose nanofibers and COF particles, as well as appreciable mechanical strength (tensile strength of 1.84 MPa and Young's modulus of 71.98 MPa), enabling their practical utilization as sorbents for gold capture from electronic waste^115^ and as electrode materials in hybrid capacitors,^116^ respectively.

**Fig. 5:**
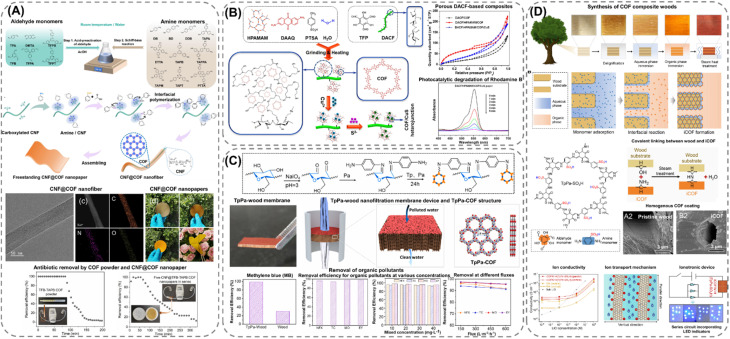
Interfacial synthetic approaches for fabricating cellulose–COF composites: (A) CNF-based freestanding membranes of imine-linked COFs synthesized *via* the interfacial growth of the COFs on the cellulose fibers at ambient conditions and in aqueous media. The membranes were fabricated through vacuum-assisted self-assembly and were utilized for the selective removal of antibiotics from aqueous media. Adapted with permission from ref. 70. Copyright 2023, American Chemical Society. (B) Composite material, consisting of dialdehyde cellulose (DACF), hyperbranched polyamide-amine (HPAMAM), and a redox-active COF (PFT-DAAQ), synthesized *via* mechanochemistry and possessing the capabilities of photocatalytically degrading organic dyes after CuS loading. Reproduced with permission from ref. 117. Copyright 2023, Springer Nature. Wood-based composites derived from the interfacial synthesis of an (C) imine-linked COF (TpPa COF) on oxidized basswood, carried out by anchoring amine monomers onto exposed aldehyde groups on the wooden surface *via* Schiff base condensation reactions. Produced TpPa-wood membranes exhibited promising properties for the removal of organic pollutants (*e.g.*, methylene blue (MB), norfloxacin (NFX), tetracycline (TC), methyl orange (MO), and eosin Y (EY)) from water by nanofiltration. Reproduced with permission from ref 118. Copyright 2023, Elsevier. (D) An ionic 2D COF (TpPa-SO_3_H) on delignified wood, fabricated by covalently linking the ionic COF (iCOF) onto the pore surface of the wood through exposed hydroxyl- and amine-groups on the wooden substrate and COF, respectively. The formed composite structures possessed well-ordered nanochannels, facilitating ultrahigh ion-transport and enabling their applicability as ionotronic devices. Reproduced with permission from ref. 119. Copyright 2023, Elsevier.

Although the fabrication of cellulose-based COF membranes can be achieved by attaching pre-synthesized COF particles onto the polymeric fibers, interfacial synthesis offers a promising alternative pathway for constructing highly uniform composite structures. This synthetic approach was demonstrated by Kong *et al.*^70^ for the fabrication of freestanding composite membranes *via* a facile polymerization process in water (Fig. 5A). The interfacial growth of the framework structures was achieved in successive steps by first orienting the amine monomers to the carboxylate groups on the modified cellulose nanofibers *via* hydrogen bonding and electrostatic interactions. Once attached, the polymerization was carried out by introducing the aldehyde monomers, resulting in a continuous growth of the COF structure along the cellulose strands. The uniform and flexible composite membranes, formed *via* vacuum-assisted self-assembly, exhibited surface areas up to 550 m^2^ g^−1^ with COF loadings ranging from 54 to 64 wt%, and demonstrated promising potential for antibiotic removal by membrane separation. Following a similar strategy, Zhang *et al.*^117^ fabricated a COF-composite paper by grafting a redox-active COF onto dialdehyde cellulose fibers (DACF) *via* a hyperbranched polyamide-amine (HPAMAM) polymer using mechanochemistry (Fig. 5B). The presence of HPAMAM served a dual role, acting both as a solid template for controlling the growth of the COF particles and as a crosslinking agent capable of forming imine linkages with the aldehyde groups on DACF. By stabilizing the growth of the COF along the cellulose fibers, a higher loading of the framework material was achieved in the composite paper compared to when HPAMAM was omitted, allowing for the subsequent loading of CuS and the utilization of the paper for the photocatalytic degradation of organic dyes. Furthermore, by extending the interfacial synthesis from 1D polymers to 3D natively structured substrates, such as wood,^118–120^ Zhang *et al.*^118^ and Fang *et al.*^119^ developed a series of robust composites from the *in situ* growth of β-ketoenamine-linked COFs on the pore surface of oxidized or delignified wood, respectively (Fig. 5C and D). Homogeneous growth of the framework structures on the wooden substrates was achieved by covalently attaching the organic monomers or COF particles *via* exposed amine, aldehyde, and hydroxy groups from the respective components, resulting in chemically and hydrolytically stable composites. Furthermore, by inheriting the intrinsic properties of the wooden substrate and the framework materials, the composites exhibited a hierarchical pore structure, which endowed them with efficient ion and molecular transport properties – crucial features for the utilization of wood-based composites in nanofiltration, storage, as well as ionotronic applications.

**Table 1:** Summary of biobased POFs and their porosity^a^

POF class	Material	Linkage	Biobased monomer	Surface area (m^2^ g^−1^)	Pore size (nm)	Pore volume their (cm^3^ g^−1^)	Ref.
HCPs	CCPOP^b^	Alkyl	Catechol	34	2.4	0.05	83
	NTPOP^b^		2,3-Naphthalene diol	93	2.0	0.22	
	MCPOP^b^		4-Methyl catechol	666	0.7	0.42	
	HCP1^c^		*o*-Methoxyphenol	14	6.6	0.02	84
	HCP2^c^		*m*-Methoxyphenol	21	6.4	0.03	
	HCP3^c^		*p*-Methoxyphenol	17	9.1	0.04	
	HCP4^c^		4-Ethyl phenol	247	3.7	0.23	
	HCP5^c^		4-Ethyl-2-methoxyphenol	15	9.2	0.04	
	HCP6^c^		4-Methyl-2-methoxyphenol	41	7.3	0.07	
	LHCPs^d^		Organosolv lignin	1076–1500	3.9–5.5	1.05–2.07	86
	OL-HCs^e^		Organosolv lignin	253*	—	0.08*	87
	Lignin-HCPs^f^		Technical lignin	2–7	<30	0.003–0.03	88
	CNSL-HCPs^g^		Cardanol and cardol	28–93	1.8–2.4	0.6–0.15	90
	pQ^h^		Quercetin	1322	<2.0	1.05	93
	pTa^h^		Tannic acid	610	<1.5	0.48	
	pBe^h^		Bark extract	1349	<2.0	1.84	
	Lignin-HCPs^f^	Aryl	Technical lignin	3–1447	<30	0.006–1.55	88
	LAPPs^i^		Technical lignin	464–1144	3.4–6.7	0.23–0.45	89
	LIGOPD-COPs^j^	Aminal	Natural lignin	15–31	8.6–200	—	85
POPs	FOFs^k^	Imine	2,5-Furandicarboxaldehyde	96–830	>0.6	0.32–2.10	75
	BIO^l^		2,5-Furandicarboxaldehyde	19	20.3	—	78
	PAN-1^m^	Aminal	2-Furanaldehyde	702	0.68	0.89	76
	FFP^n^		2,5-Furandicarboxaldehyde	773	6.4	1.24	77
	DPP^n^		Divanillin	514	3.4	0.44	
	Fb-POF^o^	Amide	2,5-Furandicarboxylic acid	208	6.4	0.57	79
	EA-TAPB and EA-TAPT^p^	Azo	Ellagic acid	196–445	1.1–12.9	0.18–0.63	95
CTFs	CTF^q^	Imine	Vanillin-derived	378	3.1	—	80
COFs	eCOFs^r^	Ester	Ellagic acid	14–35	5.6–12.9	0.08–0.19	92
	R-TPC^s^		Ellagic acid	—	1.1–1.3	—	91
	H-TPC^s^		Quercetin	—	1.1–1.4	—	
	EPCo-COF^t^	Dioxin	Ellagic acid	174	1.5	—	96
	NUS-71 and NUS-72^u^	Boronate	Ellagic acid	582–720	1.5–2.9	0.32–0.68	97

a POFs prepared from different materials.

b CCPOP: catechol, NTPOP: 2,3-naphthalene diol, and MCPOP: 4-methyl catechol crosslinked with formaldehyde dimethyl acetal (FDA).

c HCP1: *o*-methoxyphenol, HCP2: *m*-methoxyphenol, HCP3: *p*-methoxyphenol, HCP4: 4-ethyl phenol, HCP5: 4-ethyl-2-methoxyphenol, HCP6: 4-methyl-2-methoxyphenol, crosslinked with formaldehyde dimethyl acetal (FDA).

d Crosslinking of a lignin-based copolymer (LCPs), prepared from organosolv lignin, divinylbenzene (DVB), 4-vinylbenzyl chloride (VBC), and 2,2-azobis (isobutyronitrile) (AIBN), with dichloroethane (DCE).

e Organosolv lignin crosslinked with formaldehyde dimethyl acetal (FDA).

f Technical lignin, obtained from corn stalk, crosslinked with 1,4-dichloroxylene (DCX), 4,4′-bis(chloromethyl)-1,1′-biphenyl (BCMBP), or formaldehyde dimethyl acetal (FDA).

g Crosslinking of cardanol or cardol with dichloromethane.

h pQ: quercetin, pTa: tannic acid, and pBe: Bark (Pinus strobus) extract.

i Corncob lignin crosslinked with LAPP-1: 1,4-dichloroxylene and LAPP-2: 4,4′-bis(chloromethyl)-1,1′-biphenyl (BCMBP).

j Natural lignin crosslinked with 1,2-diamonobenzene (OPD) in the presence of paraformaldehyde (PFA), ammonium persulfate (APS), and p-toluene sulfonamide (PTSA).

k 2,5-diformylfuran and FOF-1: *m*-phenylenediamine, FOF-2: *p*-phenylenediamine, and FOF-3: 2,6-diaminopyridine.

l 2,5-furandicarboxaldehyde and benzene-1,3,5-triamine trihydrochloride (TAB).

m 2-Furanaldehyde and melamine.

n FFP: 2,5-furandicarboxaldehyde and melamine, and DPP: divanillin and melamine.

o 2,5-furandicarboxylic acid and melamine.

p Ellagic acid and EA-TAPB: tris(4-aminophenyl)-benzene (TAPB) or EA-TAPT: tris(4-aminophenyl)-triazine (TAPT).

q 4,4′,4″–((1,3,5-triazine-2,4,6-triyl)tris(oxy))tris(3-methoxybenzaldehyde) and *p*-phenylenediamine.

r Ellagic acid and eCOF_1_: 1,4-phenylene diisocyanate (PDI) or eCOF_2_: methylene diphenyl diisocyanate (MDI).

s Terephthaloyl chloride (TPC) and R-TPC: ellagic acid or H-TPC: quercetin.

t Ellagic acid and perfluorinated metallophthalocyanines (M = Co, Cu, or Ni).

u Ellagic acid and NUS-71: 1,3,5-benzenetriboronic acid (BTBA) or NUS-72: 1,3,5-benzenetris(4-phenylboronic acid) (BTPA). *Surface area and pore volume calculated by Grand Canonical Monte Carlo (GCMC)method from CO_2_ adsorption isotherm recorded at 0 °C.

The versatile properties of biopolymers make them highly promising for engineering difficult-to-process materials into well-defined macroscopic structures with improved properties for specific applications. By acting as both a binder and an active component, composite structures of high durability, flexibility, and functionality can be constructed, bringing these materials one step closer to practical utilization.

### Perspectives and outlook

The utilization of biopolymers for the fabrication of POFs and POF-based composites has garnered significant attention over the last decade, as the field of materials science has shifted towards more sustainable functional materials. The vast library of structurally and chemically diverse polymers not only serve as a renewable source of binder materials but also a versatile and abundant feedstock for the fabrication of functional organic monomers. A majority of studies have focused on polyphenolic and aldehyde monomers derived from biomass, which have successfully yielded fully or partially biobased POFs. Although multifunctional aromatic amines, such as methylated divanillylamine, 3,4-dimethoyxdianiline, and 4,4′-methylenebis(5-isopropyl-2-methylaniline),^121,122^ can be derived from lignin or cashew nut shell, and possess many functional and structural features required for molecular building blocks. The presence of bulky groups and aliphatic side chains in the molecules imparts steric hindrance and conformational flexibility that may obstruct the formation of POFs. Furthermore, the synthetic pathway for many aromatic amines is often complex, leading to high manufacturing costs, low yields, and scalability issues that have restricted their commercialization and widespread use. The utilization of biobased amine monomers, therefore, remains largely unexplored and has limited the development of fully biobased imine-linked POPs and COFs, in particular. Consequently, the synthesis and implementation of biobased monomers remain in their infancy. Nevertheless, we hope that this perspective may contribute to ongoing efforts and promote further development of biobased POFs and POF-composites.

Compared with monomers derived from fossil fuel feedstock, the synthesis of biobased monomers for POF construction presents several key challenges. In particular, few monomers exhibit both the desired structural property (*e.g.*, aromatic and rigid molecular structure) and chemical functionality (*e.g.*, multiple reactive sites and electron-rich or redox-active cores, such as porphyrin and anthraquinone units) that are necessary for developing highly porous and functional framework structures. Furthermore, the preparation route for multifunctional organic monomers from biomass is often complex, involving multiple chemical steps and strict synthetic procedures at harsh conditions, which not only reduces the overall yield and environmental footprint of the synthesis but also leads to increased costs. The development of crystalline POFs, in particular, faces additional challenges due to the flexible and asymmetric structure of many biobased monomers, which hinder the formation of well-ordered frameworks during polymerization and crystallization. The conformational flexibility of the monomers, therefore, often makes them better suited for the preparation of amorphous POFs. However, achieving high porosity in such materials remains a challenge that must be addressed through careful monomer selection and network design. Overall, the industrial-scale production and standardization of biobased monomers are underdeveloped, limiting their availability for both academic research and commercial applications. Addressing these challenges related to the synthesis of organic monomers and POFs is therefore critical for advancing the development of next-generation sustainable and functional materials.

The pursuit of biobased POFs and fully biobased POF-composite materials represents an exciting frontier. However, moving beyond proof-of-concept demonstrations will require integrated strategies for monomer sourcing, green synthesis, composite structuring, and life-cycle considerations. This comprehensive approach holds promise for advancing materials chemistry while also contributing to global objectives regarding resource efficiency, waste minimization, and carbon neutrality. In particular, we believe future research could focus on the following specific directions. First, expanding the library of renewable monomers is essential. While compounds such as DFF, vanillin, and ellagic acid have shown great promise, many biomass-derived aromatic and heterocyclic compounds remain vastly unexplored. The development of efficient extraction, catalytic upgrading, and functionalization methods is required to diversify the pool of available organic monomers. Furthermore, the integration of biotechnological approaches, including engineered microbial systems for targeted monomer production, may provide a powerful pathway that can accelerate this process. Second, integrating green chemistry principles and scaling-up methodologies will be pivotal to ensuring alignment with circular economy objectives. The synthesis of POFs should prioritize an aqueous and energy-efficient approach, free of hazardous organic solvents. In particular, mechanochemical synthesis and low-temperature catalytic routes are promising but require optimization to improve reproducibility during upscaling. Furthermore, comprehensive life-cycle assessments will be necessary to validate the environmental benefits of these approaches compared to conventional methodologies. Third, the advanced structural engineering of biobased POFs with biopolymers offers a facile pathway for enhancing both the materials' performance and functionality. Utilizing bio-inspired hierarchical designs, such as mimicking plant cell walls or nacre-like structures, and post-synthetic chemical crosslinking procedures can lead to improved mechanical strengths, durability, and mass transport properties in these composite structures. Furthermore, *in situ* framework growth of POFs within biopolymer matrices, combined with structuring techniques such as 3D printing, could enable the fabrication of tailored and bespoke geometries, such as microfluidic devices and catalytic reactors. Fourth, application-driven development and performance benchmarking will be crucial for transitioning the utilization of biobased POFs and POF-composites from proof-of-concept to real-life practical application. Rigorous testing under realistic operating conditions, such as continuous-flow adsorption, electrocatalysis, or membrane separation, will provide critical insight into performance stability, recyclability, and regeneration. These data will not only be essential to verify the robustness of the materials but also provide a foundation for techno-economic analyses and for industrial scale-up. Fifth, careful assessment of the full life cycle of these materials will be essential to ensure their sustainability. Biobased monomers can substantially reduce greenhouse gas emissions, manufacturing costs, and overall environmental impact compared to fossil-fuel-derived alternatives.^123–125^ However, their true sustainability is highly dependent on the biomass source, processing pathway, and end-of-life handling.^126,127^ Insufficient consideration of any of these stages can compromise environmental performance and ultimately undermine the benefits of biobased materials. To ensure valid sustainability, we believe that region-specific biomass sourced from local waste or residual streams—such as those generated by pulp and paper mills, agricultural operations, or craft industries—should serve as the primary raw material. Such sourcing minimizes transportation emissions, avoids additional land-use pressures (*e.g.*, soil degradation and biodiversity loss), and creates economic incentives for waste valorisation. Equally important is the development of optimized, integrated processes for converting biomass into multifunctional organic monomers. Improving reaction efficiency through reduced solvent and reagent use, lower energy demands, and minimized waste generation will be essential for delivering high monomer yields with a smaller environmental footprint. Furthermore, ideally, POFs should be chemically depolymerized into their constituent monomers and recycled. When recycling is not feasible, materials should be repurposed into value-added products such as functional porous carbons.

Strategies such as chemical recycling, dynamic covalent chemistry for POF disassembly, and biodegradability testing of POF-composites should therefore be integrated early in the design process. Such efforts will ensure closed-loop utilization of POFs and POF-composites but also minimize waste generation. Finally, translating laboratory advances into viable technologies will require close collaboration between academic and industrial partners. Standardized protocols for monomer production, POF synthesis, and performance evaluation can greatly accelerate the path from discovery to deployment, facilitating the utilization of biobased POF and POF-composites in real-world applications.

## Conclusions

Biomass-derived POFs and their composites represent a transformative approach toward achieving both functional performance and environmental sustainability in porous material science. By utilizing the intrinsic chemical richness of renewable feedstocks and the structural versatility of biopolymers, it is possible to address long-standing challenges related to synthetic scalability, processability, and end-use integration. The examples highlighted in this perspective demonstrate the feasibility of constructing a high-performance framework from green precursors and engineering them into freestanding, robust, and functional bulk materials.

The future of this field will depend on interdisciplinary efforts across synthetic chemistry, materials engineering, process intensification, and life-cycle assessment. Innovation in monomer production, green synthesis methods, and composite processing is expected to bring these materials closer to industrial adoption. Ultimately, a circular approach to POF design where production, application, and end-of-life are considered as a connected loop has the potential to contribute significantly to a sustainable materials economy. This approach could reduce dependence on fossil resources while enabling advanced technologies in energy storage, environmental remediation, and life science.

## Author contributions

All authors contributed equally to the writing of this work.

## Conflicts of interest

There are no conflicts to declare.

## Data Availability

No primary research results, software or code have been included and no new data were generated or analysed as part of this perspective article.
